# Unified mechanism of local drivers in a percolation model of atrial fibrillation

**DOI:** 10.1103/PhysRevE.100.062406

**Published:** 2019-12-01

**Authors:** Max Falkenberg, Andrew J. Ford, Anthony C. Li, Robert Lawrence, Alberto Ciacci, Nicholas S. Peters, Kim Christensen

**Affiliations:** 1 Blackett Laboratory, Imperial College London, London SW7 2AZ, United Kingdom; 2 Centre for Complexity Science, Imperial College London, London SW7 2AZ, United Kingdom; 3 Centre for Cardiac Engineering, Imperial College London, London W12 0NN, United Kingdom; 4 National Heart & Lung Institute, Imperial College London, London, W12 0NN, United Kingdom

## Abstract

The mechanisms of atrial fibrillation (AF) are poorly understood, resulting in disappointing success rates of ablative treatment. Different mechanisms defined largely by different atrial activation patterns have been proposed and, arguably, this dispute has slowed the progress of AF research. Recent clinical evidence suggests a unifying mechanism of local drivers based on sustained reentrant circuits in the complex atrial architecture. Here, we present a percolation inspired computational model showing spontaneous emergence of AF that strongly supports, and gives a theoretical explanation for, the clinically observed diversity of activation. We show that the difference in surface activation patterns is a direct consequence of the thickness of the discrete network of heart muscle cells through which electrical signals percolate to reach the imaged surface. The model naturally follows the clinical spectrum of AF spanning sinus rhythm, paroxysmal AF, and persistent AF as the decoupling of myocardial cells results in the lattice approaching the percolation threshold. This allows the model to make the prediction that, for paroxysmal AF, reentrant circuits emerge near the endocardium, but in persistent AF they emerge deeper in the bulk of the atrial wall. If experimentally verified, this may go towards explaining the lowering ablation success rate as AF becomes more persistent.

Atrial fibrillation (AF) is the most common cardiac arrhythmia [1], but our understanding of the underlying mechanisms is still poor [2–9]. There is growing evidence in favor of local drivers as a sustaining mechanism of AF [4,9–18]. However, there is debate about the mechanistic origin of local drivers: Some studies have identified the drivers as rotors (reentrant drivers) [12,13], whereas others have identified the drivers as focal points [11].

Recently, a study with “the potential to unify some of the previous discrepant observations” [19] has suggested that both focal and reentrant drivers may be explained by the presence of small reentrant circuits [14,20]. Using high-resolution simultaneous optical mapping of the endocardium (inner heart wall) and epicardium (outer heart wall) in explanted, diseased human hearts, the study shows that stable transmural reentrant circuits may project differently onto the endo- and epicardium. Projections typically appear as rotational activity on the endocardium and focal (breakthrough) points on the epicardium. Hence, the apparently incompatible two-dimensional (2D) projections onto the atrial walls are consistent with a single underlying mechanism of localized reentrant circuits in the transmural region [14–16].

Leading clinicians have called for further work “to confirm and extend these observations” [19]. In this paper, we develop a percolation based model of AF initiation in pursuit of this goal. Our aim is to understand the origin of the AF mechanism, how it changes as AF becomes more or less persistent, and how this effects the success rate of ablation. We take a physics approach to modeling, focusing on the essential features of the phenomenon as opposed to the fine details. This is much simpler than more biophysically realistic models and allows for concrete predictions based on large scale statistics not accessible in a laboratory setting.

The model generates activation wavefronts that propagate in a three-dimensional (3D) medium mimicking the complex discrete fiber structure of the atria. AF emerges spontaneously via the formation of spatially stable but temporally intermittent 3D reentrant circuits when the fiber network decouples, e.g., through fibrosis or fatty infiltration [21]. The model predicts that these circuits should have a minimum length of 12.5 mm, comparable with the 15 mm quoted in Ref. [14]. Reentrant circuits are quasi-one-dimensional with isolated fibers that have a width of around 2 mm, making their identification with clinically available mapping technologies difficult [22].

Our results demonstrate how 3D reentrant circuits emerge and how they are distributed in the heart wall when the coupling between cells is lowered, closely matching key clinical observations in Ref. [14]. The observed activation patterns fundamentally change as a function of the depth from the imaged surface to the driving reentrant circuit. If this depth is large, we observe focal (breakthrough) activity. If this depth is small, we observe reentrant or rotational activity.

The model predicts that the drivers of paroxysmal AF are located near the endocardium whereas the drivers of persistent AF are uniformly distributed through the atrial wall. Consistent with clinical experience, the former are more easily ablated than the latter. We test this by numerically simulating focal ablation. We find that ablation lesions that do not penetrate the full depth into the tissue are significantly less likely to destroy a reentrant circuit in persistent AF than in paroxysmal AF. Additionally, we show that in persistent AF, even if ablation successfully destroys a local source, the existence of multiple coexisting circuits prevents the global termination of AF.

## I Model

Electrical signals in the heart are mediated by discrete heart cells arranged in long, intertwined fibers. This motivates the construction of a 3D lattice model where each node acts as a single cell (or block of cells), and nodes are connected stochastically to their neighbors to mimic the branching structure of discrete heart muscle fibers, generalizing a 2D model [23]. Our model simulates the initiation and maintenance of AF from local microanatomical reentrant circuits. We do not investigate local drivers linked to cardiomyocyte automaticity, or the maintenance of fibrillation through the formation of rotors or scroll waves. Such phenomena are best studied using continuous reaction-diffusion models of cardiac electrophysiology [24].

Discrete models of cardiac tissue are well established historically and have recently regained popularity [23,25–32]. In these models, the onset of reentry has been associated with the approach from above of the bond occupation probability to the percolation threshold [29]. Some models have been extended to three dimensions to highlight the importance of the thickness of the atrial wall to the probability of reentry [30]. Specifically when investigating the role of fibrosis in cardiac arrhythmia, which is the approach taken here, there have been suggestions that discrete models are preferable over continuous models [33]. Note, mechanistically it is important to distinguish between 3D vortices in a continuous substrate which exhibit functional reentry and 3D reentrant circuits where electrically isolated fibers exhibit structural reentry. The clinical results in Ref. [14] concern structural reentrant circuits and not the wider literature on rotors and scroll waves [24,34].

We consider a simplified *L_x_* × *L_y_* × *L_z_* pipe topology of the atria with open boundaries in *x* and *z*, and periodic boundaries in *y*. Nodes are connected longitudinally to their neighbors in the *x* direction with spatial frequency *ν*_∥_ and transversely, in the *y* and *z* directions, with frequency *ν*_⊥_. Once the network is defined at the start of a simulation, it is fixed for the remainder of the simulation.

**Fig. 1:**
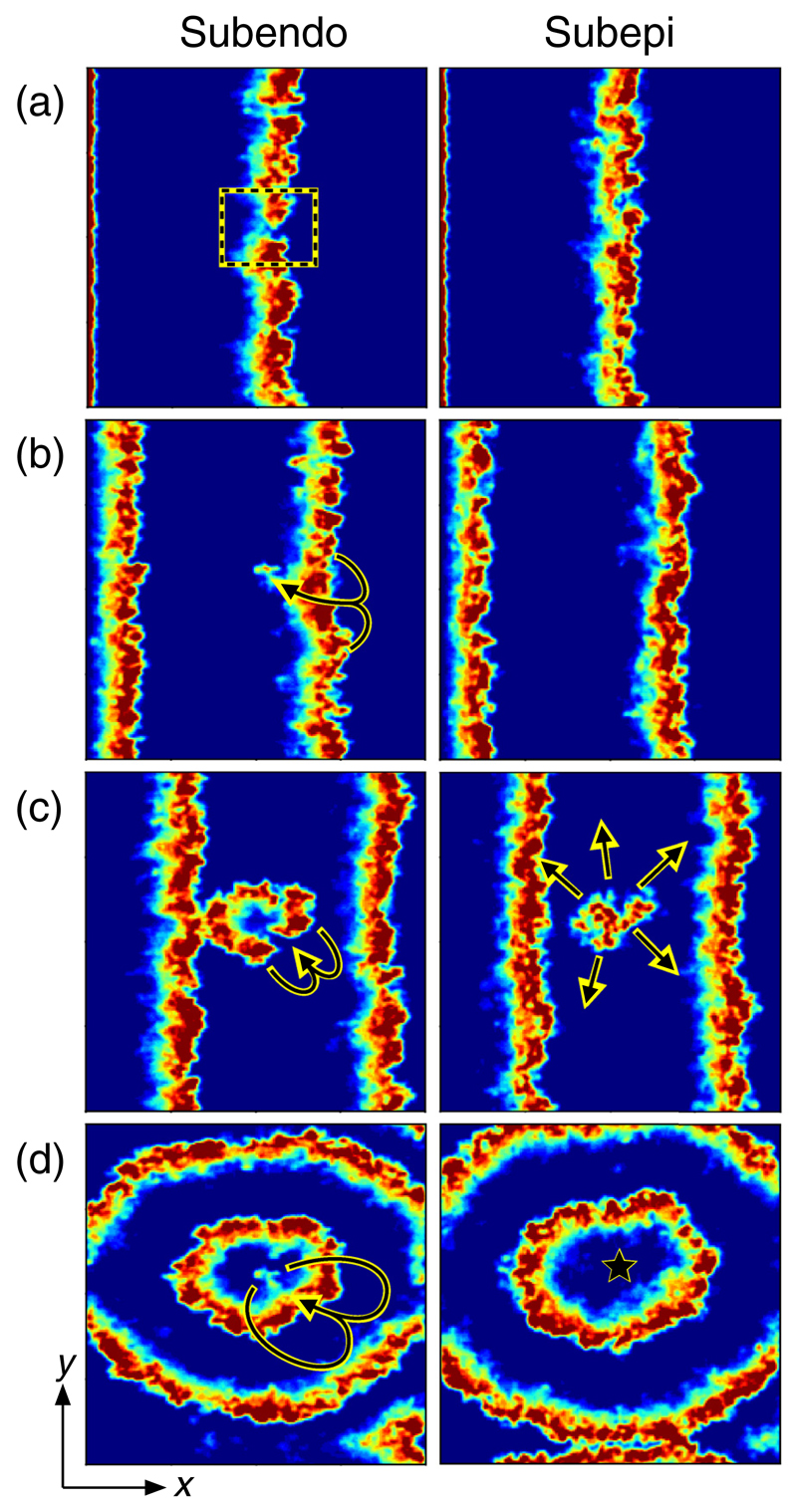
The emergence of AF in the inhomogeneous model with simultaneous endo- (*z* = 0) and epicardial (*z* = 24) imaging. Smoothing has been applied for clarity. Red, excited nodes; blue, resting (excitable) nodes; other, refractory (unexcitable) nodes. (a) Planar wavefronts propagating during sinus rhythm. The dotted box indicates a gap in the wavefront formed by conduction block. (b) Endo view: The arrow indicates an excitation reentering the gap in the wavefront. Epi view: Reentry is not observed. (c) Emergence of fibrillatory activity. (d) Maintenance of fibrillatory activity. Epi view: Activity emerges on the surface as a point source (breakthrough activity) located at the star. See video A in Supplemental Material [37].

Each node takes one of three states: resting, where the node can be excited by an active neighbor; excited; or refractory, where for *τ* time steps after excitation a node cannot be reexcited. We define the sinus node as nodes at the boundary, *x* = 0, which excites every *T* time steps. Model parameters *L_x_* = *L_y_* = 200, *L_z_* = 25, *T* = 220, and *τ* = 50 are informed by clinically observed values (see Appendix A). The model results are robust against changes in these parameters. A small fraction of nodes, *δ*, are susceptible to conduction block. With probability *ϵ*, these nodes fail to activate when their neighbors excite. When the sinus node excites in normal conduction, wavefronts are initiated at *x* = 0 and propagate smoothly in the +*x* direction and terminate at *x* = *L_x_*. However, reentrant circuits can form when nodes are sufficiently decoupled, through fibrosis [29,35], or otherwise, such that the shortest closed loop from a node back to itself is partially isolated from the remaining tissue, and the path length, in units of propagation time, exceeds the refractory period *τ*. The formation of these reentrant circuits drives fibrillatory activity [23,36] (see Fig. 1). Further examples are shown in Appendix D including figures showing close-up examples of reentrant circuits and focal breakthrough points. Videos of typical activation patterns are included in the Supplemental Material [37] and are captioned in Appendix E.

**Fig. 2:**
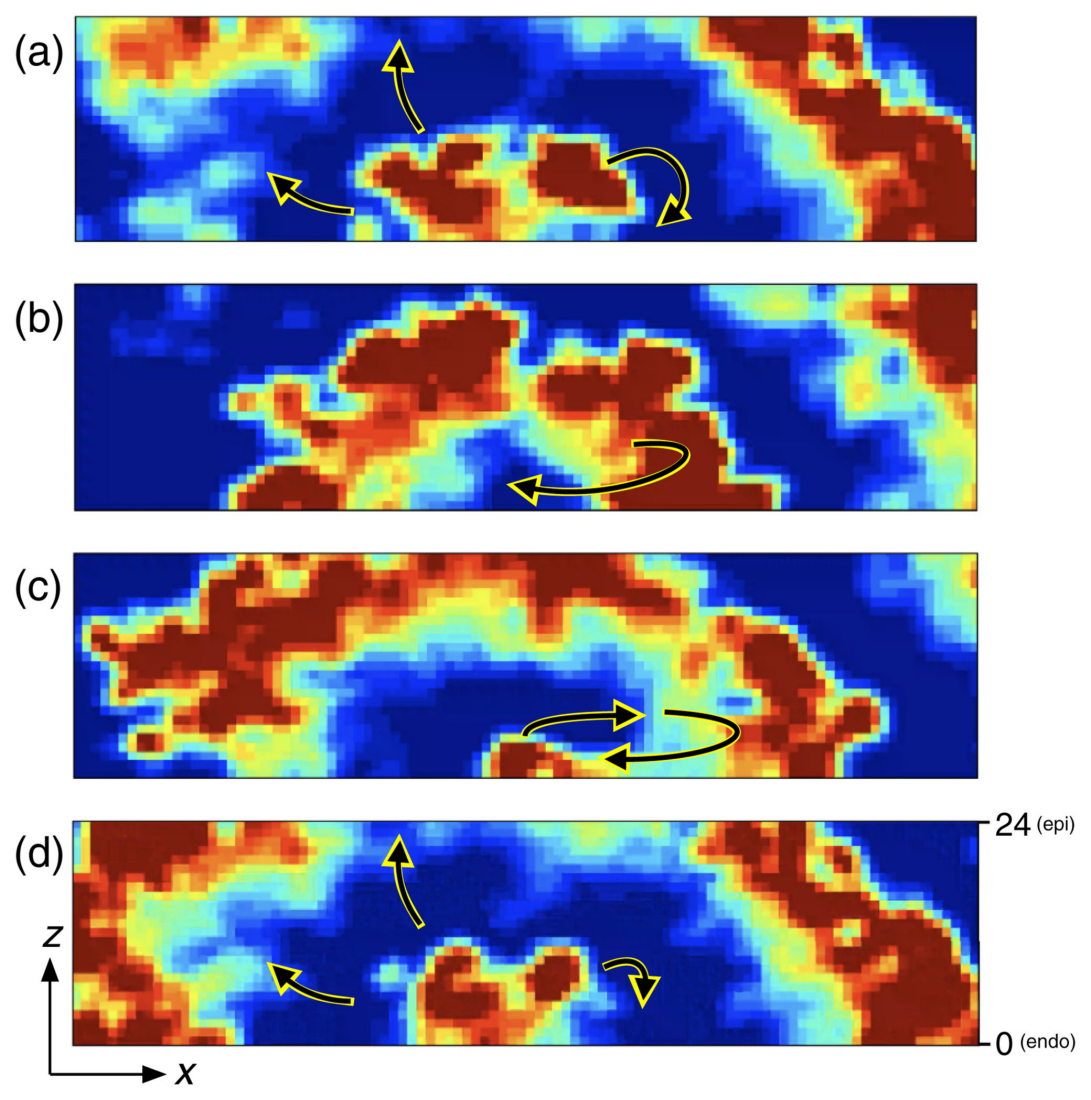
A cross section of the model during AF showing a closeup of the *x*-*z* plane (fixed *y* coordinate). The cross section has been chosen to align with a reentrant circuit. Arrows indicate the movement of wavefronts. The top (bottom) surface of each panel corresponds to the epicardium (endocardium). A reentrant circuit can be seen near the endocardium with activity (a) moving around an isolated fiber, (b, c) reentering the isolated fiber, and (d) emitting fibrillatory waves from the left end of the isolated fiber. Fibrillatory activity spreads from the endocardium to the epicardium through the complex fiber structure of the model. This results in a wide variety of possible breakthrough patterns on the epicardial surface. When viewed from the endocardium, reentrant activity will be clearly visible since the isolated fiber lies along the endocardial surface. When viewed from the epicardial surface, the wavefronts propagating from the reentrant circuit may emerge as a single breakthrough point, or as multiple breakthrough points simultaneously. See video D in Supplemental Material [37]. Scale: 100 × 25 nodes.

Reference [14] finds that reentry activity is typically observed on the endocardial surface, whereas focal points are often found on the epicardial surface. Additionally, there is much stronger longitudinal coupling on the endo- than the epicardial surface. To test whether fiber orientation can account for the distribution of activation patterns observed clinically, we consider a homogeneous and inhomogeneous model. In the homogeneous model all nodes are connected to their neighbors with the same frequencies, *ν*_∥_ and *ν*_⊥_. In the inhomogeneous model, the variation in fiber direction changes with depth. Here we fix the average decoupling of nodes, $\bar{\nu }$ = (2*ν*_∥_ + 4*ν*_⊥_)*/*6, and vary linearly the average fiber angle, Δ*θ* = tan^-1^ (*ν*_⊥_/*ν*_∥_), in each layer from Δ*θ*_endo_ = 24° at the endocardium (*z* = 0) to Δ*θ*_epi_ = 42° at the epicardium (*z* = 24). These values are taken from clinical data [16]. The introduction of fiber inhomogeneity into the model results in reentrant circuits preferentially anchoring to the endocardium. An example of this can be seen in Fig. 2, which shows a cross section of the model activation patterns from the endocardium to the epicardium. A reentrant circuit is shown anchored to the endocardium, with the activation wavefronts propagating from one end of the circuit to the epicardial surface and emerging as a focal breakthrough point.

To study the effect of local ablation in the model, we identify the first location at which a reentrant circuit forms. A new simulation with the identical structure is generated and the nodes at the identified location are destroyed, mimicking focal ablation, up to a prespecified depth (see Appendix C for details). This process is repeated until the locations of the first ten identified reentrant circuits are ablated. This allows us to study (a) the *global* success rate of ablation as a function of $\bar{\nu }$ by measuring the probability that ablation prevents AF emerging anywhere in the tissue and (b) the *local* success rate of ablation as a function of the ablation depth *z*, by measuring the probability that an ablation, that does not penetrate the full depth into the tissue, destroys the reentrant circuit at a given location.

## II Results

**Fig. 3:**
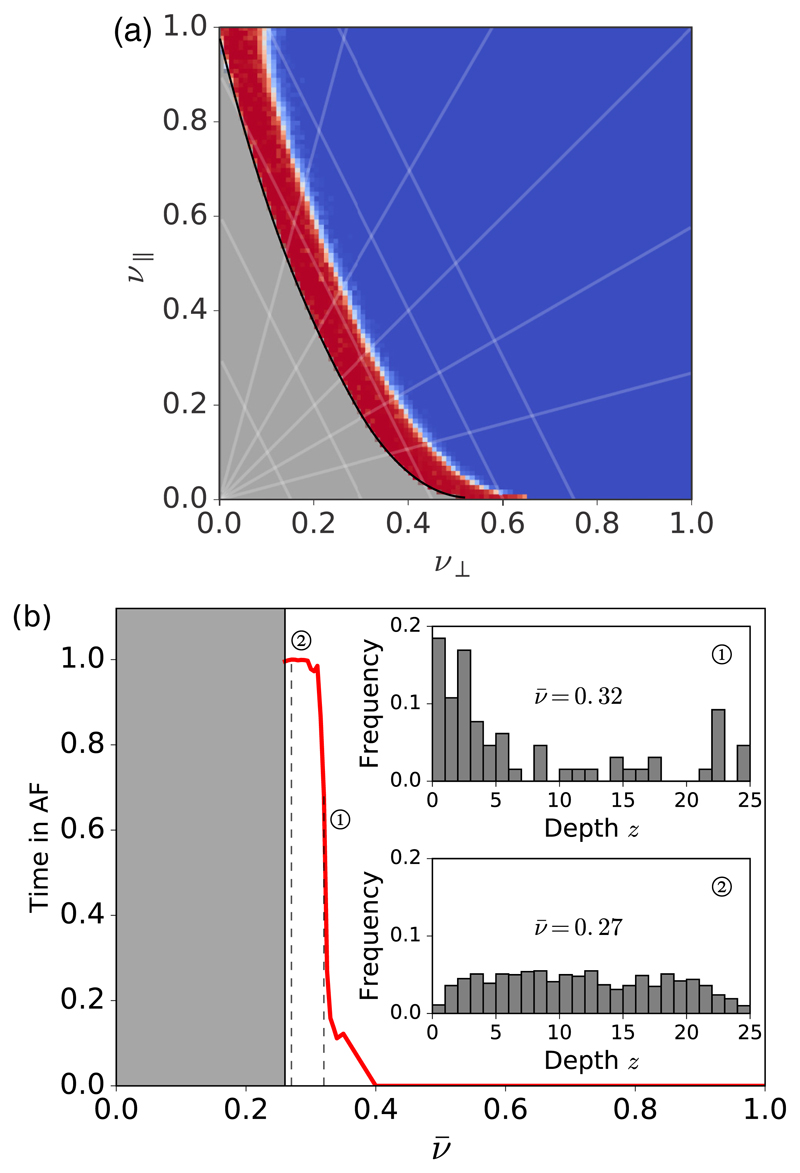
The average time spent in AF for the (a) homogeneous and (b) inhomogeneous models. Both models show a transition from sinus rhythm at large coupling (0% AF time) through paroxysmal AF (0% < AF time < 100%) to persistent AF (100% AF time). The gray parameter regions indicate coupling parameters below the percolation threshold, and are thus irrelevant to clinical AF. (a) Homogeneous coupling parameters, *ν*_∥_ and *ν*_⊥_. Blue, sinus rhythm; red, persistent AF. The white transition region corresponds to paroxysmal AF. Guidelines of positive (negative) gradient indicate constant Δ*θ* ($\bar{\nu }$). Risk curve as a function of the inhomogenous coupling parameter, $\bar{\nu }$, (red graph). There is no AF for $\bar{\nu }$ ≳ 0.4. Decreasing $\bar{\nu }$ associated with decoupling nodes, there is a transition from sinus rhythm through paroxysmal AF to persistent AF. Inset: Histograms showing the distribution of reentrant circuits driving AF for paroxysmal AF (top) and persistent AF (bottom) as a function of depth *z* from the endocardium. Drivers cluster in the subendocardial region (*z* = 0) for paroxysmal AF but are uniformly distributed across the bulk for persistent AF.

The phase spaces for the risk of entering AF for the homogeneous and inhomogeneous models are shown in Fig. 3. For large values of the coupling parameters (no fibrosis) the model exhibits sinus rhythm indefinitely. As the coupling reduces, a small number of reentrant circuits can form. Here we observe paroxysmal AF with intermittent episodes of irregular activity. As the coupling is even further reduced, the model enters persistent AF where, once AF has been initiated, the model will never return to sinus rhythm without external intervention. These results are consistent with recent evidence showing that local drivers anchor at or near fibrotic lesions [13], with fibrosis increasing the number of reentrant regions and the time spent in AF [38].

In the homogeneous model the endo- and epicardium are equivalent, and we find that in paroxysmal AF the majority of drivers form equivalently on either surface, whereas for persistent AF the majority of drivers form in the bulk and not on the surfaces (see Fig. 16 in Appendix D).

The inhomogeneous model breaks the symmetry between the endo- and epicardium. Here we find that in paroxysmal AF drivers form preferentially near the endocardial surface, with very few drivers forming on the epicardial surface and almost none in the bulk of the atria [see Fig. 3(b), inset 1]. However, in persistent AF, drivers are uniformly distributed throughout the atrial wall [see Fig. 3(b), inset 2]. Hence, as AF becomes more persistent, the average position of drivers moves away from the endocardium and into the bulk of the atrial wall. The variation in fiber orientation is the only asymmetry between the endo- and epicardium and is therefore responsible for the asymmetry in the reentrant circuit depths. Because the method by which reentrant circuits form is fully local [39], the presence of an isolated fiber at one point in the tissue is independent of the tissue elsewhere in the model. Therefore, the results are robust against different choices of fiber orientation and tissue thickness that may be associated with the left or right atria [40], and show that reentrant circuits will first emerge on the surface with the strongest longitudinal coupling (see Figs. 17 and 19 in Appendix D).

**Fig. 4:**
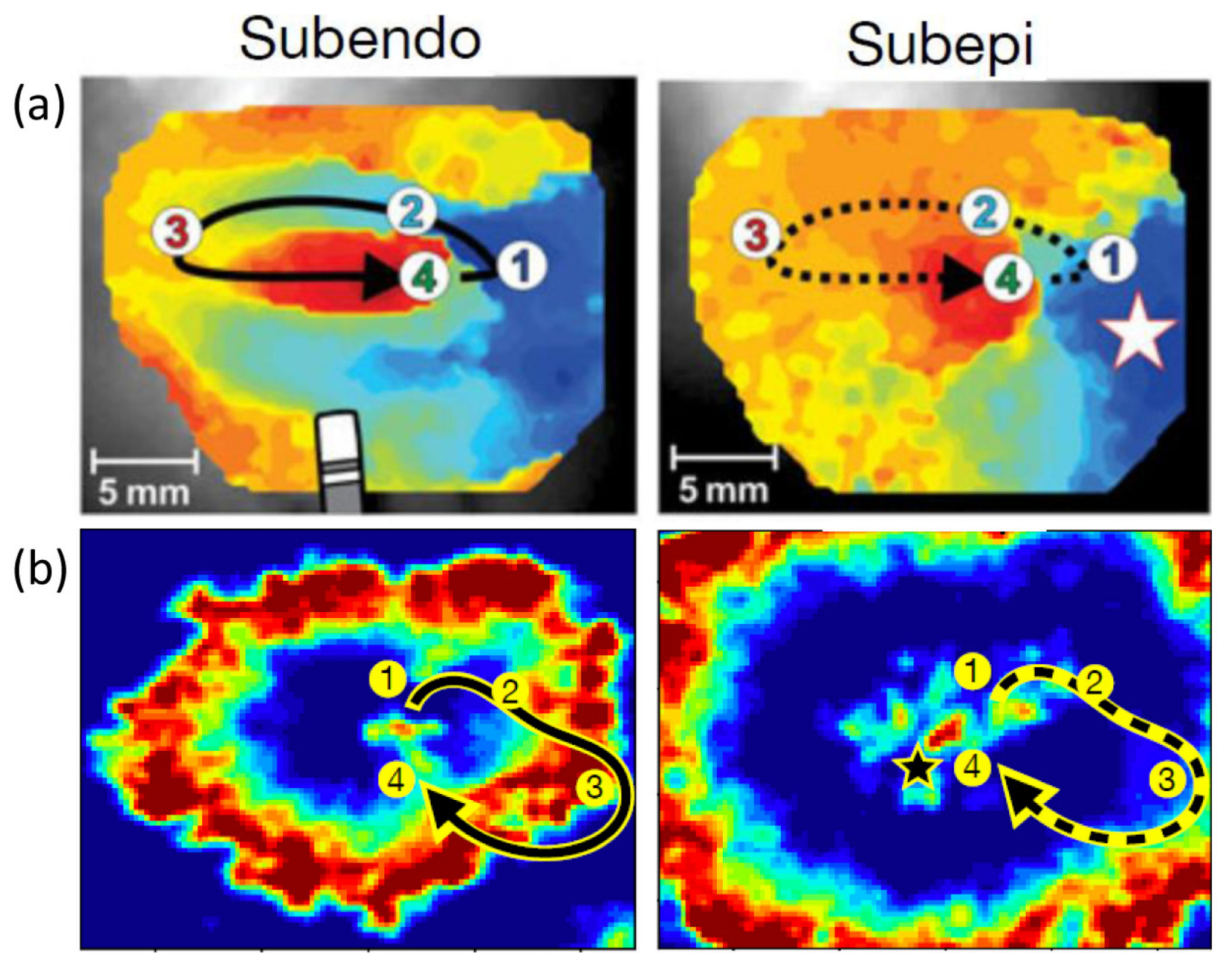
(a) An activation map adapted with permission from Ref. [14] showing reentry activity from subendo imaging and a focal source on the subepi side with four marked reference points. (b) The equivalent for our model. Smoothing has been applied to images for clarity. Red, excited nodes; blue, resting nodes; other, refractory nodes.

Visualizing the activation patterns in the inhomogeneous model on the surfaces at *z* = 0 (endocardium) and *z* = 24 (epicardium), we observe activation patterns consistent with Ref. [14]. In Fig. 4, the left (right) panel shows the activity when viewed from the endocardium (epicardium). For the activation maps, electrical activity spreads across the tissue from point 1 through point 2, but is blocked from reaching the isolated fiber at point 4. The activity loops around the isolated fiber and reenters the fiber at point 3. This back-propagating excitation passes through point 4 before reexciting point 1, which sustains the reentrant circuit. Viewing the same region from the epicardium does not show the same reentry activity. Instead, the excitation emerges as a point source indicated by a star. The surface activity driven from the atrial bulk will typically appear as a breakthrough point, as opposed to reentry activity. A cross section of the activation patterns from endo- to epicardium is shown in Fig. 2.

**Fig. 5:**
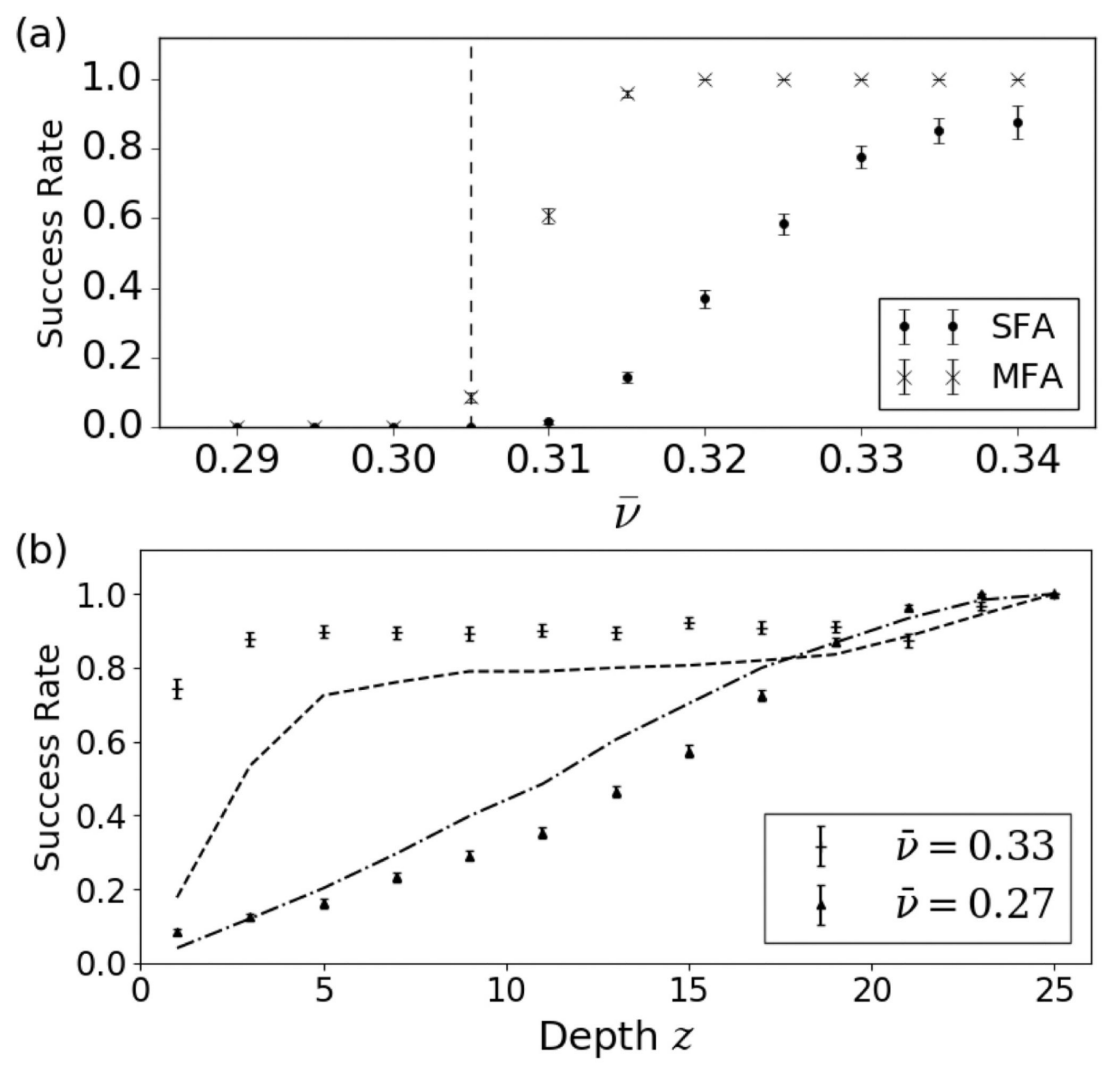
(a) The global success rate of full depth focal ablation if the first reentrant circuit location is destroyed [single focal ablation (SFA), circles], and if the first ten locations are destroyed [multiple focal ablations (MFA), crosses] as a function of the coupling parameter, $\bar{\nu }$. The dashed line indicates the coupling value below which all simulated tissues harbour reentrant circuits. (b) The local success rate of a single focal ablation destroying reentrant circuits at the ablation site as a function of the ablation depth, *z*, for tissues with high coupling, $\bar{\nu }$ = 0.33 (plus), and low coupling, $\bar{\nu }$ = 0.27 (up triangle). The dashed (dot-dashed) lines correspond to the cumulative sum of the reentrant circuit depth probabilities for $\bar{\nu }$ = 0.33 ($\bar{\nu }$ = 0.27).

**Fig. 6:**
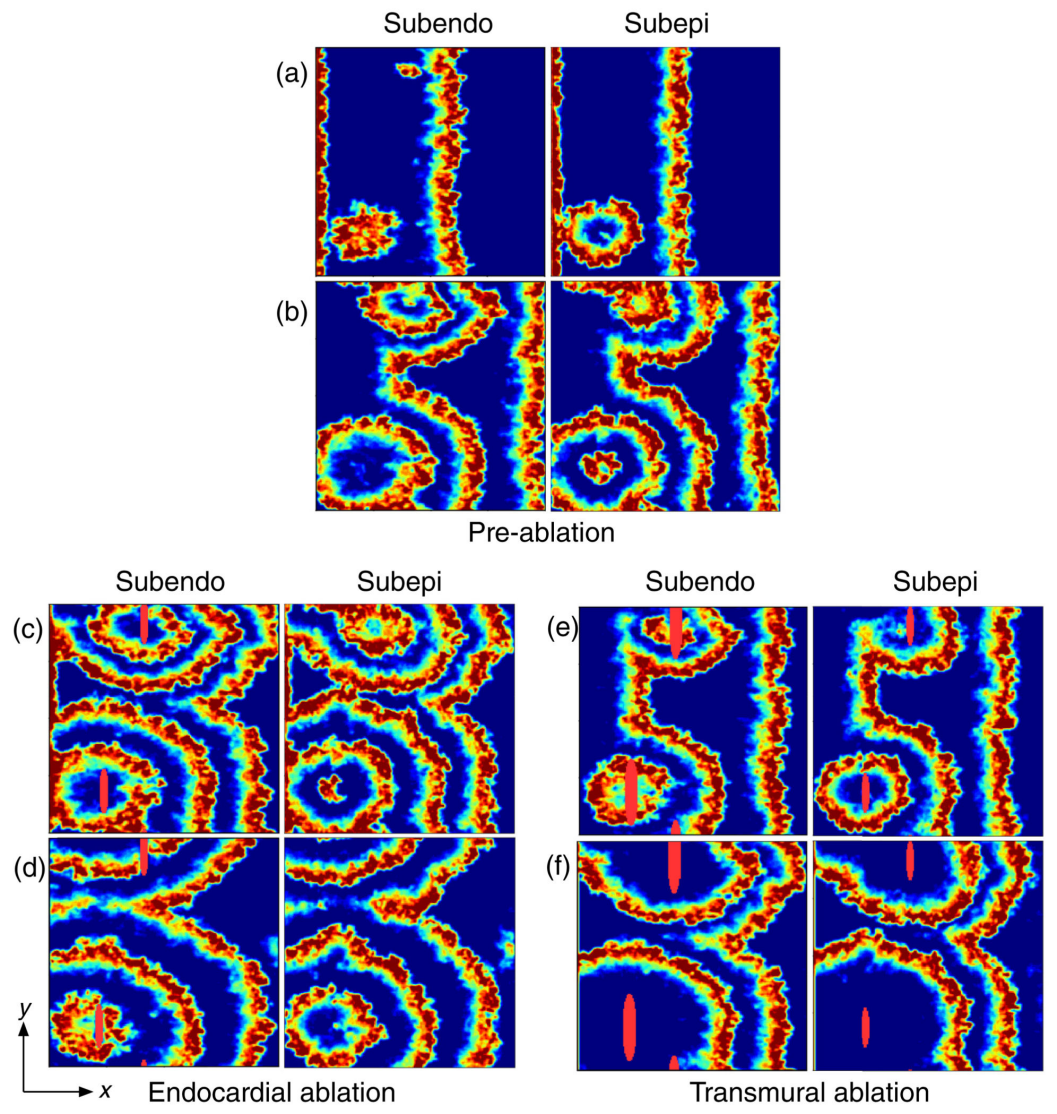
Simulated ablation in a tissue with multiple drivers demonstrating how the ability to terminate fibrillatory activity is closely related to driver depth. AF emerges (a) in the bottom left corner from a reentrant circuit near the epicardium, and (b) at the top of the tissue from a separate reentrant circuit near the endocardium. (c) Two shallow ablations (solid red in bottom left and top center) are applied to the endocardial surface at the locations of the reentrant circuits penetrating part of the way into the tissue. (d) The circuit near the epicardium has not been destroyed by the ablation since the ablation has not penetrated far enough into the tissue. The circuit near the endocardium has been destroyed successfully. However, since an active circuit remains in the tissue, AF has not been terminated. (e) and (f) show the same ablation process as demonstrated in (c) and (d); however, the ablation is now transmural, penetrating the full distance through the tissue. This is indicated by the solid red regions on the subepi view in (e) and (f). The ablations have successfully destroyed both reentrant circuits. Hence, fibrillatory waves dissipate as shown in (f) and sinus rhythm will be restored. Note, transmural ablation is not always possible in clinical practice. Ablation lesions typically struggle to penetrate more than 2 mm [41] into the atrial wall, which can be up to 7 mm thick in places [14]. Scale: 200 × 200 nodes (full tissue).

Figure 5 shows the global and local success rate of ablation in the model. Globally, Fig. 5(a) indicates that the success of ablation exhibits phase transition-like behavior. At low coupling, focal ablation consistently fails to terminate fibrillation, independent of the number of ablation lesions. Above a transition coupling value, the ablation success rate rapidly increases with increased coupling. The success rate is higher if multiple ablations are applied. The results indicate that focal ablation is increasingly ineffective as AF becomes more persistent. However, despite the fact that ablation fails globally, Fig. 5(b) indicates that, locally, ablation does successfully destroy the targeted reentrant circuits if the ablation is transmural. For nontransmural ablation, ablation has a much higher local success rate at high coupling values—half depth ablation has a success rate of 90% (50%) at high (low) coupling. A direct comparison between transmural and nontransmural ablation is shown in Fig. 6. The activation maps shown demonstrate that for reentrant circuits anchored to the endocardium a shallow ablation is sufficient to destroy the reentrant circuit. However, for reentrant circuits anchored to the epicardium, nontransmural ablation may fail to destroy the reentrant circuit driving AF.

In Fig. 3(a), there are percolation-type transitions [42] associated with the black and white curves, respectively. The black curve outlines the bond percolation threshold for the fiber network itself: Below (above) the black curve, the fiber network does not (does) percolate from *x* = 0 to *L_x_*. This transition is irrelevant for clinical AF but the other transition is highly relevant. The white curve outlines the transition where the finite surfaces associated with missing bonds (known as hulls in three dimensions or holes in two dimensions [43]) have a linear dimension larger than or equal to *τ/*2—these hulls are needed to form an isolated fiber. A fingerprint of the latter transition is also seen in Fig. 5(a). Below (above) the vertical dashed line at $\bar{\nu }$ ≈ 0.305, the global success rate of multiple focal ablations is zero (nonzero). In an infinite lattice, this corresponds to the number of finite hulls with linear dimension > *τ/*2 being infinite (finite) slightly below (above) this threshold.

## III Discussion

These model observations are directly compatible with recent clinical findings that identified rotational activity from endocardial mapping in patients with paroxysmal AF [12,39], and another clinical observation identifying focal activity in patients with persistent AF from epicardial mapping [11]. Moreover, our model offers a natural explanation why ablation is more successful for paroxysmal than persistent AF. Studies have shown that ablation lesions become smaller as they penetrate further into the tissue from the endocardial surface, and that ablation struggles to penetrate more than 2 mm into the atrial wall [41], which can be up to 7 mm thick [14]. Hence, because the average driver position moves deeper into the atrial wall as AF becomes more persistent, ablation lesions need to be more accurately positioned and they must penetrate further into the tissue, restricting ablation efficacy. The model suggests that if the atria are sufficiently fibrotic, reentrant circuits are so numerous that focal ablation cannot terminate AF, even with multiple ablation lesions [see Fig. 5(a)].

The model may explain the prevalence of paroxysmal AF in the pulmonary veins (PVs), where sleeves of cardiac tissue extend into the PVs [20,44]. The sleeves get thinner further into the PVs and there are significant changes in the fiber orientation from the endo- to epicardium [45–47]. These physiological changes reduce cell-to-cell coupling, which the model has shown is the key requirement for forming the reentrant circuits that drive AF. Intriguingly, another recent study also demonstrates transmural reentry, specifically in the PVs, under the assumption of isotropic conduction velocities [48].

## IV Limitations And Next Steps

Our model is much simpler than alternative biophysically realistic models [24,34]. We do not account for rate dependent effects in the action potential propagation, although we expect this to play a minimal role in the development and maintenance of structural reentrant circuits. This has the benefit that we are able to perform unparalleled statistical analyses to fully explore the differences between paroxysmal and persistent AF, but at the cost of electrophysiological realism. As a result, our model focuses purely on the initiation and maintenance of AF from microanatomical reentrant circuits and not on AF initiated or maintained by other methods such as cardiomyocyte automaticity or the formation of rotors and scroll waves. These mechanisms are best studied using alternative models.

In our model, we consider general structural properties such as the difference in fiber orientation between the endo- and epicardium, but we do not explicitly account for specific anatomical fiber bundles in the real heart or differences between the left and right atria. Therefore, with the current framework, we cannot explain why local drivers are found more frequently in the left atrium than in the right atrium [10,13]. However, in a proof of concept work where we have adapted the current methods for use with a real atrial fiber map, reentrant circuits do appear to form preferentially in the left atrium [49]. Additionally, we observe circuits forming in a number of key locations suspected for their role in the formation of local drivers in persistent AF, including the sleeves of the pulmonary veins, the posterior atrial wall, and the atrial appendages [50].

To address these limitations and verify the results presented here, we propose a potential experimental test and a number of suggested model developments. First, adapting the model to a realistic atrial topology with an integrated fiber structure will allow us to refine the predictions made here and make concrete statements about different regions of the atria and specific anatomical structures. This includes investigating which atrial regions are especially prone to the formation of reentrant circuits and testing whether these correlate with known clinical data. We have taken the first steps to adapt the current model to a realistic atrial fiber structure in [49]. Currently, both the model presented here and the adapted model consider the role of local decoupling of muscle fibers, simulating the action of diffuse interstitial fibrosis or fatty infiltration. However, more pronounced global decoupling may occur in regions with compact fibrotic lesions. Future versions of the model should consider a full range of potential fibrosis patterns when analyzing the emergence of reentrant circuits.

As a potential experimental verification of our results, we propose following the methods set out in Ref. [14]. Using explanted Langendorff-perfused animal hearts with a diffuse fibrosis model at different levels of fibrotic burden, simultaneous endo- and epicardial optical mapping may be used to assess how the distribution and depth of microanatomical reentrant circuits change with increasing fibrotic burden. As the fibrotic burden is increased, our model would predict an increase in the number of observed drivers, and a reduction (increase) in the ratio of reentrant drivers to focal drivers seen on the endocardium (epicardium).

## V Conclusions

The overarching aim of this paper was to create the simplest possible bioinspired model that displays the variety of clinically observed activation patterns [14] and can exploit large scale statistics to make concrete predictions not accessible in a laboratory setting.

The model is consistent with the typical evolution of AF from sinus rhythm through paroxysmal AF to persistent AF. Moreover, combined with the clinical observations in Ref. [14], our model gives substantial evidence for the proposed unifying mechanism of local drivers in AF, suggesting a potential resolution to long-standing debates. Complementing this finding, we predict that the inhomogeneity in fiber orientation causes the average depth of drivers to move away from the endocardium and into the bulk of the atrial wall as AF becomes more persistent, potentially explaining why ablation is less successful for persistent AF than for paroxysmal AF, if experimentally verified. This insight is the first of its kind and may have key clinical implications.

Work is currently underway on adapting the model to realistic atrial fiber maps with the hope that this may be able to identify reentrant circuit risk regions clinically on a personalized basis [51,52].

## Supplementary Material

Video G

Video A Endo

Video E Epi

Video E Endo

Video D Epi

Video D Endo

Video F

Video D Cross section

Video C Endo

Video B Epi

Video B Endo

Video A Epi

Video C Epi

Appendices
