# Supplementary material for: Unified mechanism of local drivers in a percolation model of atrial fibrillation
Source: Phys Rev E. Author manuscript; Available in PMC 2020 Jun 24. (PMC7314598; doi:10.1103/PhysRevE.100.062406)
Supplement: Appendices [file EMS86645-supplement-Appendices.pdf]

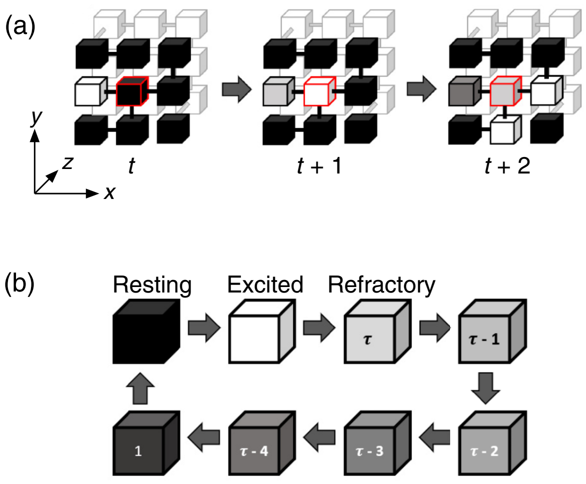

FIG. 7. (a) The excitation wave propagates across neighboring nodes. Nodes are connected longitudinally with frequency $\nu_{\parallel}$ and transversely (along y and z directions) with frequency $\nu_{\perp}$. At time t, the red bordered node is resting. At time $t+1$, the node activates in response to the stimulus coming from its neighbor to the left. At time $t+2$, the red bordered node becomes refractory and the excitation wave activates the two nodes in the resting state to which it is coupled. The node to the left of the red bordered node cannot be excited as it is in a refractory state. (b) Temporal dynamics of a node. Each node sequentially passes across three distinct states: resting (black), excited (white), and refractory (gray scale).

APPENDIX A: MODEL DEFINITION

1. Heart muscle structure

The area of the atrial muscle is $L'_x \times L'_y \approx 20\,\mathrm{cm}^2$ [53,54] while the thickness of the atrial muscle wall ranges from 1 to 7 mm [14]. A typical average is about $L'_z = 2.5$ mm [55]. The atrial muscle tissue comprises tubular shaped heart muscle cells (myocytes) of length $\Delta x' \approx 100\,\mu\mathrm{m}$ and diameter $\Delta y' = \Delta z' \approx 20\,\mu\mathrm{m}$ [56,57]. Cells are predominantly connected longitudinally, forming fibers that connect transversely now and again. Hence a branching network of anisotropic cells provides an appropriate approximation of the atrial tissue.

2. Heart muscle physiology

The membrane potential of heart muscle cells shows that they can be in one of three states: excited, where the cell depolarizes; resting, where the cell can be depolarized by a neighboring active cell; and refractory, where for some time τ' after excitation the cell cannot be reexcited. The shortest time scale is associated with a cell depolarizing, $\Delta t' \approx 0.6$ ms, whereas the refractory period lasts $\tau' \approx 150$ ms under rapid pacing. When a resting cell is induced to excite by a neighboring excited cell, it contracts. The propagation speed of the activation wavefront is $\Delta x'/\Delta t' \approx 0.2\,\mathrm{ms}^{-1}$ and the heart's natural pacemaker (sinus node) ensures the periodic propagation of a coordinated activation wavefront, causing atrial muscle contractions. The rate of excitations is controlled by the pacemaker cells that regularly self-excite with period $T' = 300$–1000 ms. For example, $T' = 660$ ms corresponds to approximately 90 beats per minute.

3. Dimensionless parameters of the model

We can translate real tissue values into dimensionless model parameters. We apply a similar conversion procedure to that presented in Ref. [23]. The dimensions of the simplified 3D topology are $L_x = L'_x/\Delta x' = 1000$, $L_y = L'_y/\Delta x' = 1000$, and $L_z = L'_z/\Delta z' = 125$. We apply a coarse grain transformation $\Delta x' \to b\Delta x'$, $\Delta y' \to b\Delta y'$, $\Delta z' \to b\Delta z'$ such that we

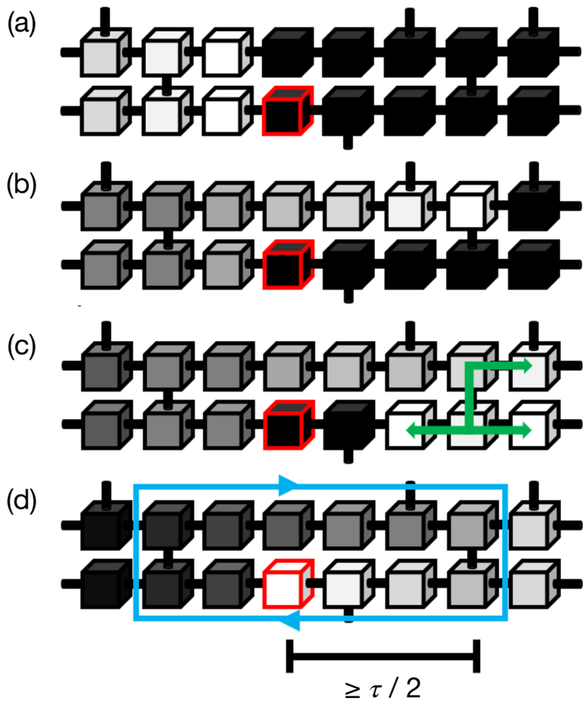

FIG. 8. Progression of the excitation wave through a simple reentrant circuit situated on the surface of the 3D topology used to mimic the heart muscle fiber network. The red bordered node is susceptible to conduction block. (a, b) The planar excitation wave reaches the node that is susceptible to conduction block. The red bordered node does not excite and blocks the propagation in the lower fiber while the planar propagation of the wavefront proceeds in the upper fiber. (b, c) The activation wavefront reaches the penultimate node in the upper fiber that connects the two adjacent fibers. The excitation advances in multiple directions, indicated by the green arrows. In particular, the wavefront reenters the fiber where the original conduction was blocked. (c, d) If the path length indicated in the lower fiber is longer than $\tau/2$, the node placed to the left of the red bordered node will have reached its resting state when the backward propagation arrives. Therefore, the region indicated in light blue will act as a reentrant circuit until the red bordered node blocks the excitation again. The likelihood of creating a reentrant circuit is determined by the coupling frequencies $\nu_\parallel$ and $\nu_\perp$ and the fraction δ of nodes susceptible to conduction block.

group b^3 cells within one node of the model. The propagation speed has to remain constant $\Delta x'/\Delta t' \approx 0.2\,\text{ms}^{-1}$ so we must also coarse grain time $\Delta t' \to b\Delta t'$ [23]. By using $b = 5$, we find the dimensionless parameters of the model. For the 3D pipe structure, we have $L_x = L'_x/(b\Delta x') = 200$, $L_y = L'_y/(b\Delta y') = 200$, and $L_z = L'_z/(b\Delta z') = 25$. The renormalized excitation time $b\Delta t' = 3\,\text{ms}$ yields a dimensionless refractory period $\tau = \tau'/(b\Delta t') = 50$ and pacemaker activation period $T = T'/(b\Delta t') = 220$, assuming $T' = 660\,\text{ms}$.

4. Modeling the real atrial tissue

The structural properties are mimicked by a 3D pipelike topology of the atria, $L_x \times L_y \times L_z$, where x is the longitudinal direction and y and z are the transverse directions. Given that the mechanism of reentrant circuit formation in the model is fully local, the precise topology chosen for the model has a

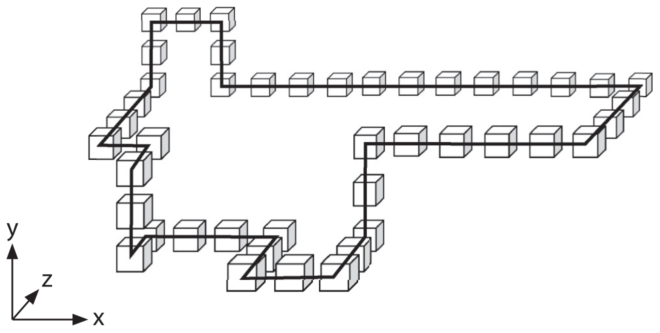

FIG. 9. Three-dimensional visualization of an AF driver located in the bulk of the atrial tissue. The black path represents the connectivity structure between the nodes. In this particular example we observe that longitudinal connections are more frequent than transverse connections (y and z directions), representative of reentrant circuits that can be observed when $\nu_\parallel$ is substantially larger than $\nu_\perp$.

minimal effect on the results. The pacemaker cells are the nodes at the boundary $x = 0$ and we apply open boundaries in x and z and periodic boundaries in y. Each of the $L_z = 25$ layers contain $L_x \times L_y = 200 \times 200$ nodes. The nodes are connected longitudinally with frequency $\nu_\parallel$ and transversely (within the layer and across the layers) with frequency $\nu_\perp$. The network of nodes mimics the branching structure of heart muscle fibers and it is kept fixed for a given realization.

To mimic the membrane potential of a cell, nodes can be in one of three distinct states: resting, excited, or refractory. At a given time t, an excited node induces resting nodes to which it is coupled to excite at time $t+1$. The excited node at time t becomes refractory at time $t+1$ and it will not be able to respond to any further stimulus during the next τ time steps.

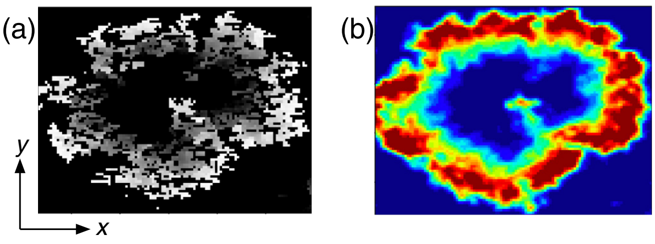

FIG. 10. Surface activation patterns in the model (a) before and (b) after processing. (a) The raw node states of a single layer of nodes in the model. White indicates active nodes, black nodes are resting (excitable), and gray nodes are refractory. This raw image only shows the node states in a single layer at a fixed z coordinate. (b) To aid data interpretation and match the visualization methods applied in Ref. [14] we process the raw image using Gaussian smoothing. A 3D Gaussian kernel is used to convolve the raw node states. The Gaussian is centered on the imaged surface with standard deviations $\sigma_x = \sigma_y = 1$ and $\sigma_z = 5$. The color scheme for the activation maps after processing has been chosen to match that used in Ref. [14]. Active wavefronts are shown in red, resting nodes are in blue, and refractory nodes are represented by all other colors. This filtered image improves the coherence of wavefronts in the model and aids data interpretation. Scale: 100×75 nodes.

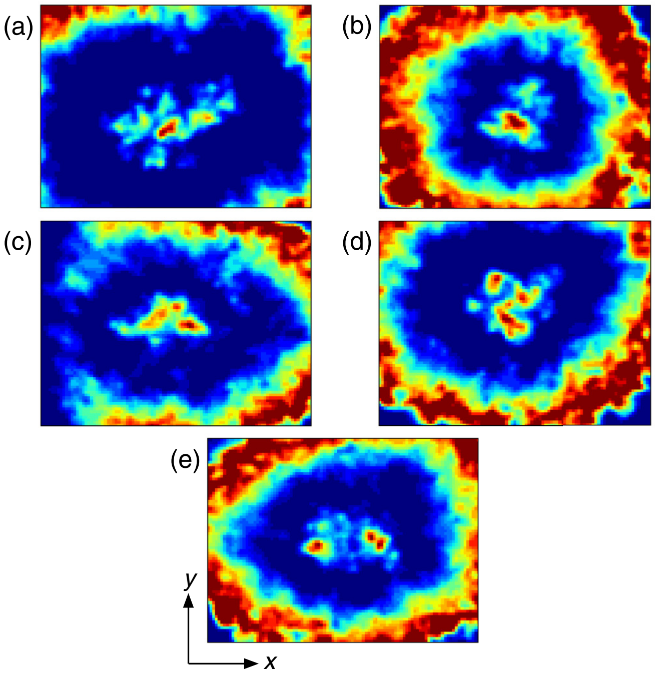

FIG. 11. Examples of breakthrough patterns in the model. Close-up view of activity for five different realizations (tissues). Tissues (a) and (b) show one primary breakthrough point. Tissues (c) and (d) show the activity emerging across a wider area almost simultaneously. Tissue (e) shows two clearly divided breakthrough points. Globally, all five tissues have breakthrough activity that will generate waves that appear to originate from a focal point. These activation patterns are typically observed on the epicardium during paroxysmal AF, but may, in rare cases, also be seen on the endocardium. Quantitatively differentiating between breakthrough types is difficult. See videos A, B, C, and E. Scale: 100×75 nodes.

The refractory node will revert to a resting node after τ time steps (see Fig. 7).

A small fraction δ of the nodes can be susceptible to conduction block. These nodes are selected at random and their position in the network is fixed for a given realization. These particular nodes will, with a small probability ϵ, fail to activate when their neighbors excite. In a well connected network, such a failure will not disrupt the planar propagation of the excitation wave and sinus rhythm prevails. However, if the network of heart muscle cells becomes too decoupled, for example due to fibrosis, then, when a failure occurs, the planar propagation of the excitation wave may be disrupted. The break of the regular wavefront may prompt the spontaneous emergence of circuits formed by wavelets of excitation leaking back through the refractory wake of the

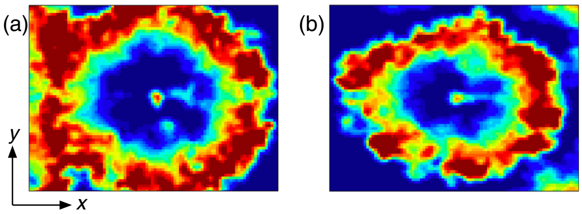

FIG. 12. Examples of partial and full reentry in the model. (a) An example of partial surface reentry where a full rotation is not observed in the surface activity. This is because the reentrant circuit stretches to a depth of approximately four nodes from the endocardial surface. (b) Complete surface reentry, where no gaps are observed in the reentrant circuit. Reentrant circuits are typically observed on the endocardium during paroxysmal AF, but may also be found on the epicardium in rare cases. See video D. Scale: 90×70 nodes.

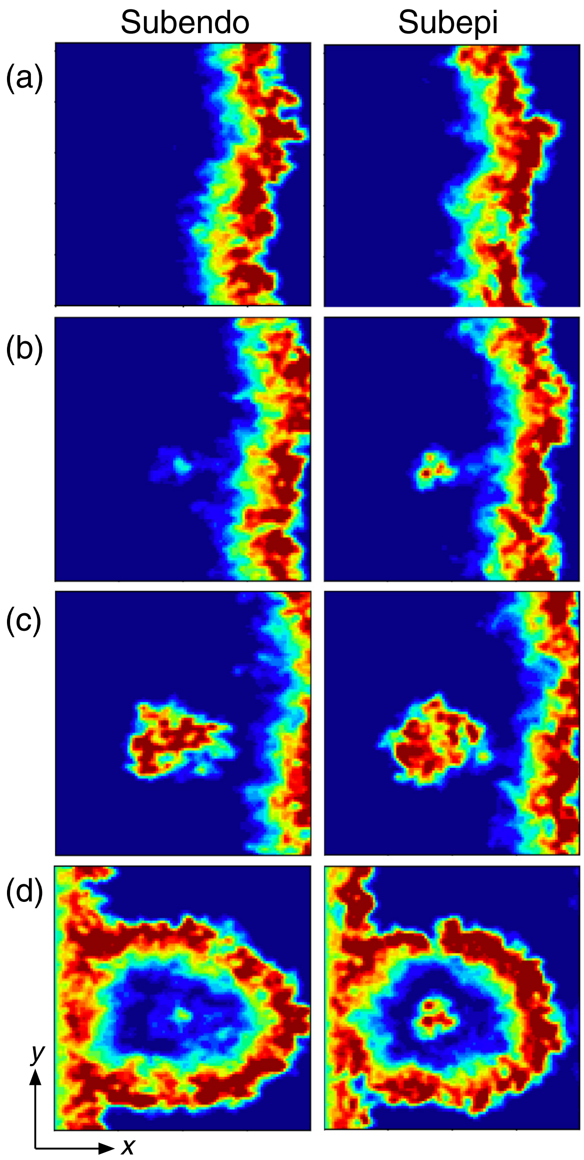

FIG. 13. An example of surface activation patterns where reentrant activity is not observed on either atrial surface. Close-up view of the activation pattern in the case of a reentrant circuit with depth $z = 16$, $\bar{\nu} = 0.31$. (a) The tissue in sinus rhythm with regularly propagating wavefronts. A signature of fibrillatory activity characterizing AF is not observed. (b) Initial fibrillatory activity emerges on both epicardium and endocardium as breakthrough points. Note that this reentrant circuit is eight layers closer to the epicardium than the endocardium, as can be seen by the delay between activation patterns. (c) Fibrillatory activity spreads across the tissue. (d) AF is sustained. Reentrant circuits are not observed on either surface. Scale: 100×100 nodes.

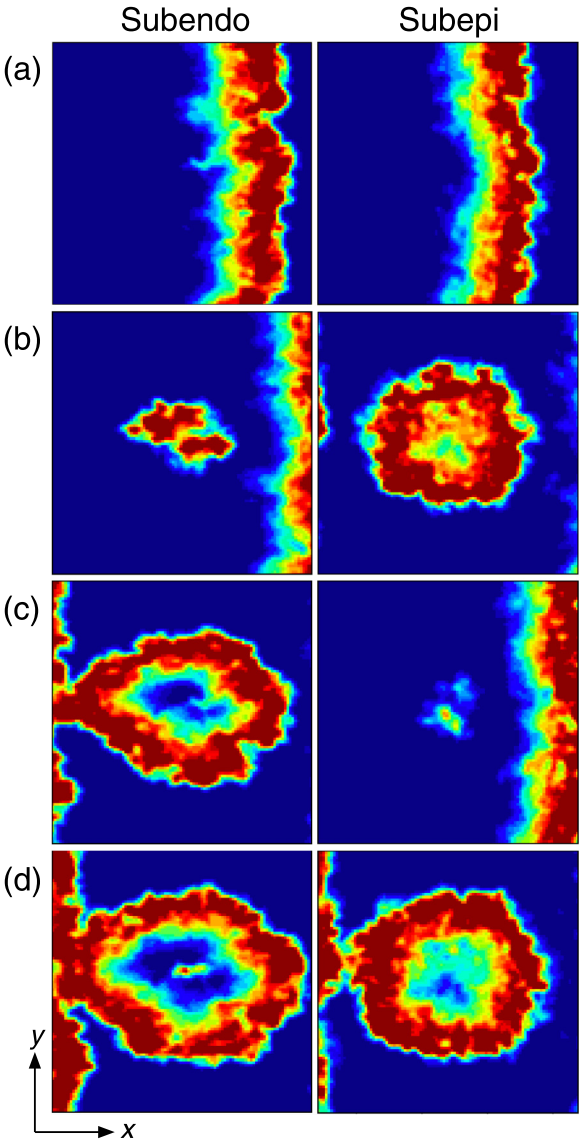

FIG. 14. An example of reentrant activity observed on the endocardial surface. Close-up view of activation patterns in the case of a reentrant circuit near the endocardial surface with depth $z = 1$, $\bar{\nu} = 0.33$. (a) The model in sinus rhythm. A faint reentrant excitation is observed at the center of the subendo view. (b) Fibrillatory activity emerges in the model. An unexcited tract on the right side of the active region in the subendo view indicates the isolated fiber for the reentrant circuit. Activity spreads on the subepi view resembling a focal source. (c) The wavefront reenters the unexcited tract in the subendo view. The activity breaks through onto the epicardial surface at a single stable breakthrough point. (d) Fibrillatory activity is sustained. Scale: 100×100 nodes.

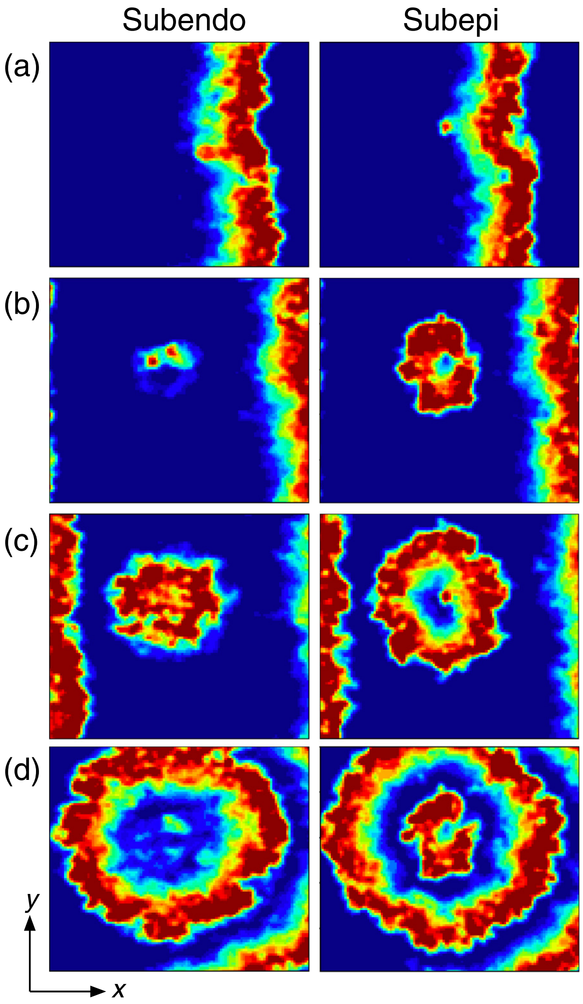

FIG. 15. An example of reentrant activity observed on the epicardial surface. Close-up view of the activation pattern in the case of a reentry circuit near the epicardium with depth $z = 22$, $\bar{\nu} = 0.32$. Circuits forming near the epicardium are much rarer than those forming near the endocardium in paroxysmal AF. (a) The model in sinus rhythm. A faint reentrant excitation is observed above the center of the subepi view. (b) Fibrillatory activity emerges in the model. An excitation is just starting to enter the unexcited tract on the right side of the active region in the subepi. Fibrillatory activity has broken through to the endocardial surface at multiple points simultaneously. (c, d) Fibrillatory activity is driven and sustained. A reentrant circuit is clearly observed from subepi imaging. Activity appears to originate from a focal point in subendo imaging. Scale: 110×100 nodes.

wavefront [23]. This occurs if nodes in a certain region of the atrial muscle form a reentrant circuit (see Fig. 8). Reentrant circuits can emerge spontaneously in the atria due to structural changes during the lifetime of a patient as specific pathologies (e.g., fibrosis) might decrease the coupling between cells.

The critical structures discussed in Fig. 8 have been introduced in Ref. [23]. In the three-dimensional network, similar configurations are relevant but now they can be placed within the bulk as well as on the endo- and epicardial surfaces (see Fig. 9 for a simple visualization).

APPENDIX B: DATA VISUALIZATION

The raw model data contain small scale noise which obscures the overall behavior. Therefore, to effectively visualize model activity, the raw data need to be filtered in a manner mimicking real clinical imaging techniques. The optical mapping system used by Ref. [14] is recorded to a depth

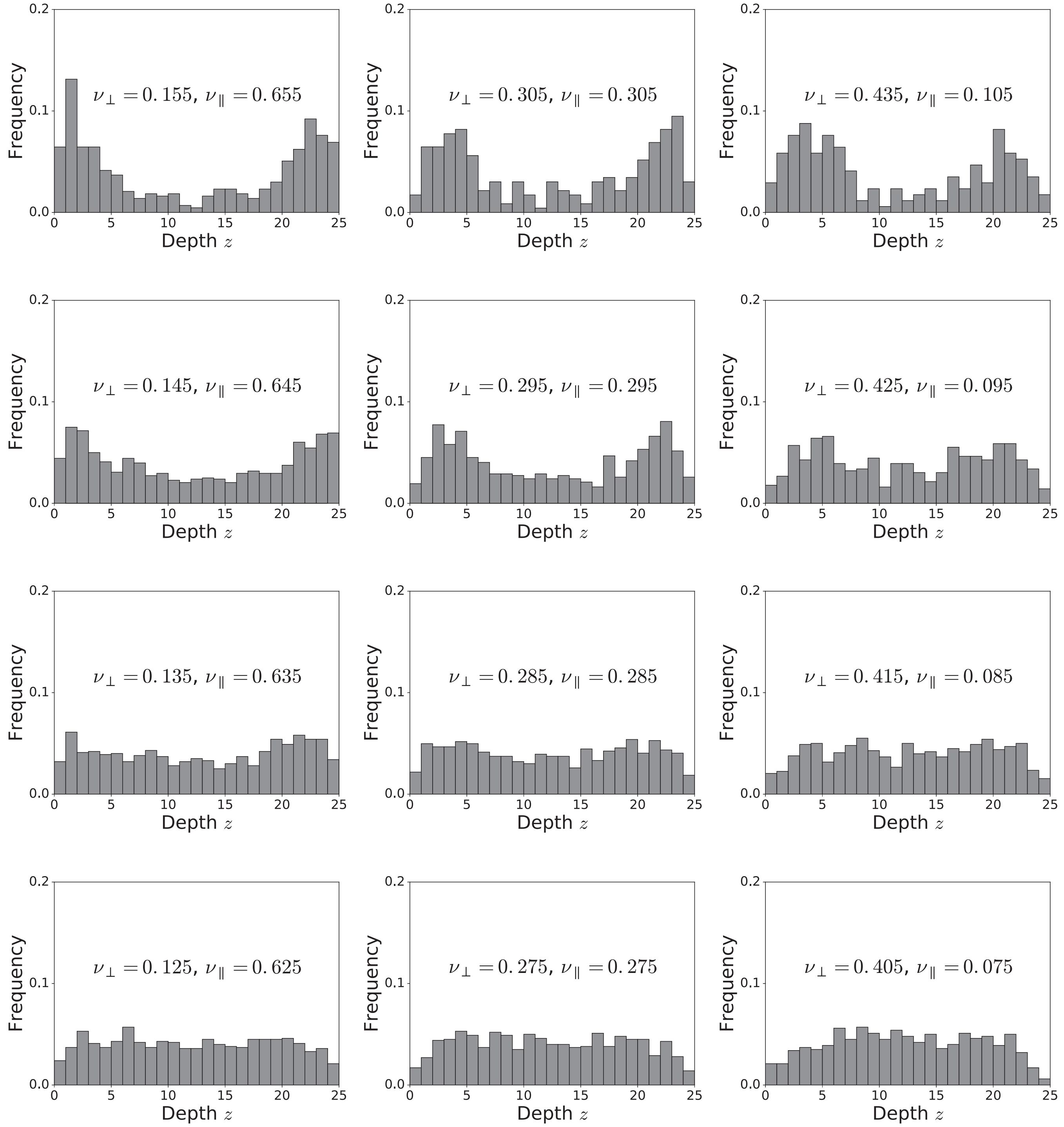

FIG. 16. The distribution of reentrant circuit depth z from the endocardium in the homogeneous model. Fiber angle $\Delta\theta$ is constant across the tissue. AF progresses from paroxysmal AF (top row) to persistent AF (bottom row). The left column is for tissues with strong longitudinal coupling and weak transverse coupling. The central column is for tissues with equal longitudinal and transverse coupling. The right column is for tissues with weak longitudinal coupling and strong transverse coupling. Depth $z = 0$ ($z = 24$) corresponds to the endocardium (epicardium). In paroxysmal AF, reentrant circuits are predominantly found near the atrial surfaces. When visualized from the atrial surface, these circuits will typically appear as reentrant or rotational activity. In persistent AF, reentrant circuits are uniformly distributed in the bulk of the atrial wall, away from the epicardium and endocardium. These circuits are more likely to result in breakthrough point (focal) activation patterns when visualized from the atrial surfaces. In the homogeneous model, the endocardium and epicardium are equivalent due to the symmetry.

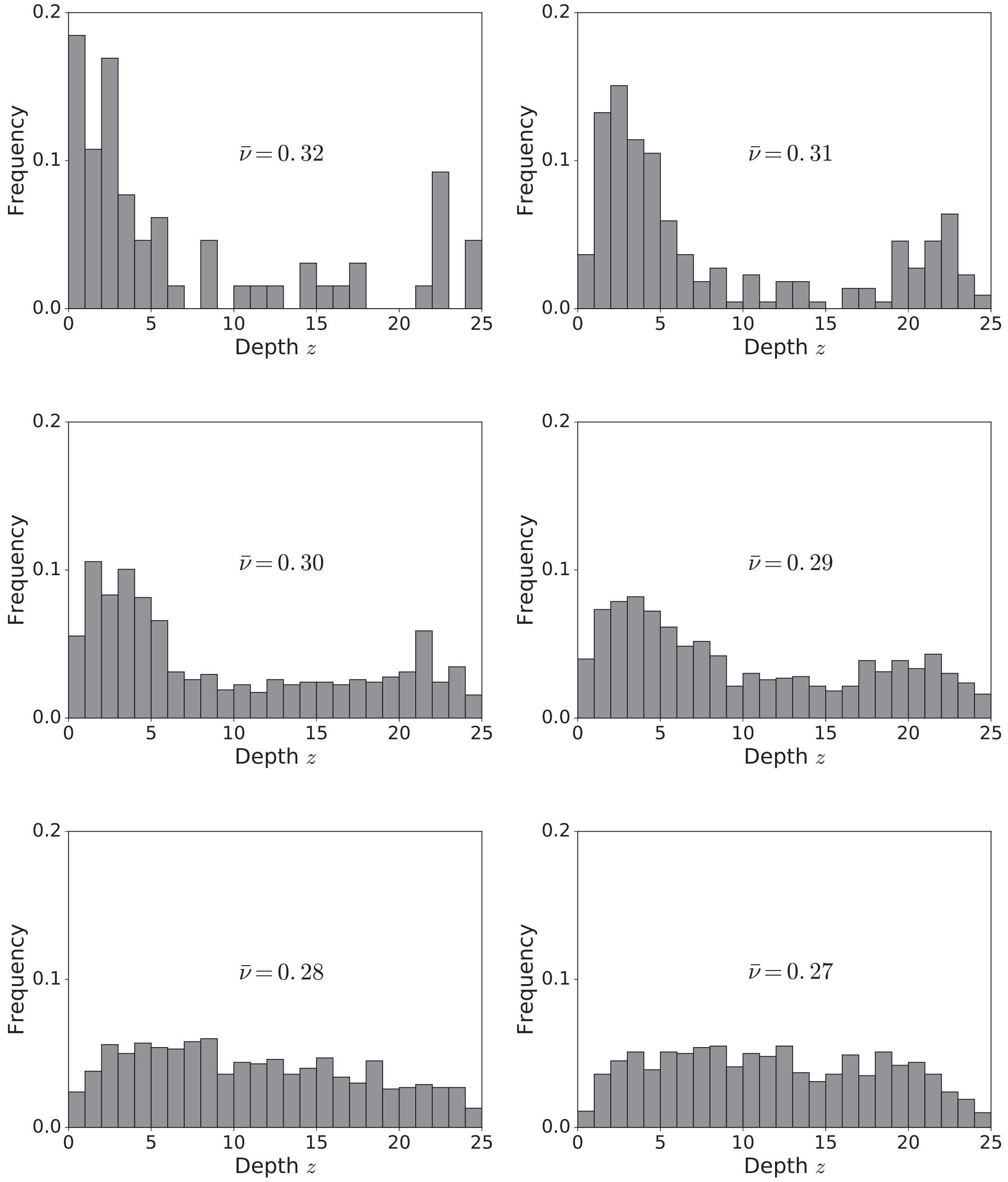

FIG. 17. The distribution of reentrant circuit depth z from the endocardium in the inhomogeneous model. AF progresses from paroxysmal AF ($\bar{\nu} = 0.32$) to persistent AF ($\bar{\nu} = 0.27$). Fiber angle varies linearly across the tissue from $\Delta\theta_{\text{endo}} = 24^\circ$ on the endocardium to $\Delta\theta_{\text{epi}} = 42^\circ$ on the epicardium. Depth $z = 0$ ($z = 24$) corresponds to the endocardium (epicardium). In the inhomogeneous model, the symmetry between the epicardium and endocardium is broken with the stronger longitudinal coupling on the endocardium than the epicardium. For paroxysmal AF, reentrant circuits are predominantly found near the atrial surfaces, and significantly more circuits cluster near the endocardium than the epicardium. This is because fibers have much stronger longitudinal coupling on the endocardial surface than on the epicardial surface. When visualized from the endocardium, these circuits will typically appear as reentrant or rotational activity. In persistent AF, reentrant circuits are uniformly distributed in the bulk of the atrial wall, away from the epicardium and endocardium. These circuits are more likely to result in breakthrough point (focal) activation patterns when visualized from the endocardium. As AF has progressed from paroxysmal to persistent, the average depth of circuits from the endocardium at $z = 0$ has significantly increased—this hinders the effective use of catheter ablation for those circuits deep in the atrial bulk.

of 1–4 mm weighted to the layer being observed, with a resolution of approximately 0.02 mm. These data guided the coarse graining: The activation patterns were convolved with a 3D Gaussian, with standard deviations $\sigma_x = \sigma_y = 1$ and $\sigma_z = 5$. An example of the difference between the raw and processed images is shown in Fig. 10.

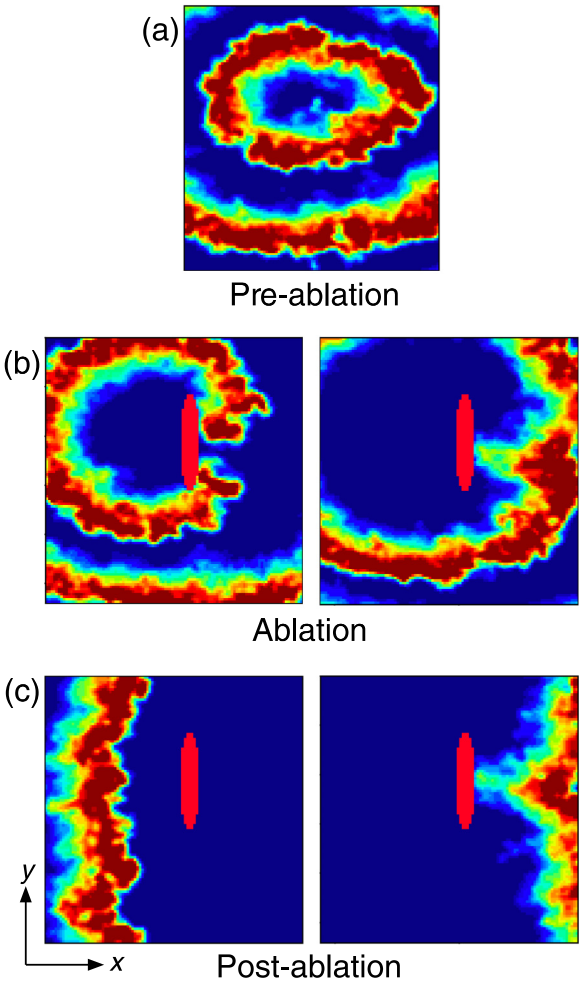

FIG. 18. The termination of AF by destroying the reentrant circuit using ablation in the inhomogeneous model. The images show the activation patterns for a close-up of the endocardial surface. (a) A faint reentrant excitation is visible just above the center of the image. This corresponds to the reentrant circuit which is driving AF. (b) Nodes surrounding the reentrant circuit are destroyed (solid red region). The reentry circuit is not sustained since the reentrant excitation collides with the ablation lesion. The fibrillatory waves dissipate. (c) The model has returned to sinus rhythm. The ablation lesion disrupts the sinus wavefront, but does not induce fibrillation. See video F. Scale: 100×110 nodes.

APPENDIX C: SIMULATION DETAILS—ABLATION METHODS

To simulate the effect of ablation in the model (results shown in Fig. 5 of the main paper) we generate 500 independent tissues for each coupling value, $\bar{\nu}$. The random seeds used to generate these tissues are saved so that the tissues can be regenerated at a later stage. An additional seed is generated for each tissue which specifies the order in which nodes susceptible to conduction block fail to excite when prompted by an excited neighbor.

For the global success rate of ablation [see Fig. 5(a) in the main paper], a simulation is started in each tissue up to a maximum time of 10 000 time steps. For simulations of length 10 000 ($\approx$45 beats), with $\epsilon = 0.05$ and $T = 220$, if a structure capable of generating a reentrant circuit is present, the probability it activates during the simulation is >90%. If a reentrant circuit is detected during the evolution of the simulation, the simulation is stopped and the location of the circuit is recorded. Otherwise, we mark this structure as containing no reentrant circuits and move on to the next. If a reentrant circuit is detected, using the saved seeds, the exact same tissue is regenerated and we reset the order in which nodes susceptible to conduction block fail to fire. Before the simulation is restarted, a cuboid of side length 20 nodes and depth 25 nodes, centered on the x and y coordinates identified as the circuit location, is destroyed, mimicking a focal ablation. For a tissue of dimensions $200 \times 200 \times 25$ nodes, this ablation lesion corresponds to 1% of the tissue. Destroyed nodes are permanently disabled and cannot be excited by neighboring excited nodes. After the ablation lesion is applied, we restart the simulation for another 10 000 time steps and observe whether another reentrant circuit emerges at a different location in the tissue. If yes, this region can be ablated and the process can be repeated an arbitrary number of times—at this stage the focal ablation has been unsuccessful at terminating AF globally. If no, the ablation is considered successful and we move onto the next tissue.

The global ablation success curves shown in Fig. 5(a) of the main paper correspond to the percentage success that ablation prevents the emergence of AF anywhere in the tissue after a single ablation has been applied, and after up to ten individual lesions have been applied.

For the local success rate of ablation [see Fig. 5(b) in the main paper], we also generate 500 random tissues for each coupling value and save their seeds. Likewise, we save the seeds giving the sequence of failures for the nodes susceptible to conduction block. For each tissue, we run a simulation for up to 10 000 time steps. If a reentrant circuit is not identified we move onto the next tissue. If a circuit is identified, a cuboid of side length 20 nodes and depth z nodes, centered on the x and y coordinates identified as the circuit location, is destroyed. Additionally, all nodes susceptible to conduction block with coordinates *outside* the x and y extent of the ablation lesion are prevented from exhibiting unidirectional conduction block. Hence, when the simulation is restarted, a reentrant circuit can only form in the region directly below the ablation lesion, that is, a circuit can only form if it is at a coordinate corresponding to the x and y range ablated, but with depth greater than that of the ablation lesion. The simulation is now restarted for 10 000 time steps and we observe whether or not a reentrant circuit is detected. If yes, the local ablation has failed. If no, the local ablation has been successful. This process is repeated for a range of ablation depths z from a single node layer to the full tissue depth $z = 25$, using identical tissues such that different depth results can be compared like for like.

There are three reasons local ablation might fail: (1) the ablation lesion is not deep enough to destroy a reentrant circuit, (2) the targeted reentrant circuit is destroyed but a different independent circuit is present below the ablation lesion, or (3) the ablation lesion itself decouples nodes such that a circuit which was not present previously can now form by anchoring to the ablation lesion itself.

APPENDIX D: FURTHER MODEL RESULTS

1. Activation maps

In the model, we observe a wide variety of surface activation maps. These can be characterized as full surface reentry (where the active wavefront is visible for the full reentry cycle), partial surface reentry (where only part of the reentry cycle is visible), single location breakthrough points, and multiple quasisimultaneous breakthrough points. The surface activity that is observed in any given tissue depends on the depth of the reentrant circuit as well as the level of connectivity in the tissue. Close-up breakthrough point activation maps are shown in Fig. 11; full and partial reentry maps are shown in Fig. 12.

For a reentrant circuit located in the bulk, both the endocardial and epicardial activation maps are similar, as shown in Fig. 13. A stronger delay can be seen for circuits on one surface. Reentrant circuits are seen on the endocardium and epicardium in Figs. 14 and 15, respectively. Breakthrough points are observed on the surface opposite the reentrant circuit.

2. Depth of reentrant circuits

We can measure the depth from the endocardium (z coordinate) at which reentrant circuits form in the homogeneous and inhomogeneous model. Figure 16 (Fig. 17) shows the distribution of reentrant circuit depths for the homogeneous (inhomogeneous) model at various points on the model's phase space, shown in Fig. 3(a) [Fig. 3(b)] in the main paper. Note, reentrant circuits have a spatial extent and are not only present at a single coordinate in the tissue. However, for simplicity, the algorithm to identify reentrant circuits only identifies the breakthrough point of the isolated fiber in a reentrant circuit, corresponding to point 1 in the activation maps shown in Fig. 4 of the main paper.

In the homogeneous model, the fiber structure is statistically equivalent across the whole tissue. Hence, there is no distinction between the endocardial surface at $z = 0$ and the epicardial surface at $z = 24$. Figure 16 shows that, in paroxysmal AF, reentrant circuits form preferentially near the atrial surfaces. As nodes continue to decouple and the model approaches persistent AF, reentrant circuits form uniformly in the bulk. This dependence of reentrant circuit depth on AF persistence is found to exist for models with strong longitudinal and weak transverse coupling (Fig. 16 left column), equal longitudinal and transverse coupling (Fig. 16 central column), and weak longitudinal and transverse coupling (Fig. 16 right column). However, reentrant circuits cluster around the atrial surfaces more closely for strong longitudinal and weak transverse coupling than for weak longitudinal and strong transverse coupling.

As a result, when the fiber orientation is varied in the inhomogeneous model, reentrant circuits will form preferentially in the regions with the strongest longitudinal coupling. This breaks the symmetry in reentrant circuit distribution between the endocardium and the epicardium in the inhomogeneous model. In the inhomogeneous model for paroxysmal AF, reentrant circuits are found to cluster preferentially at the atrial surfaces, as opposed to uniformly in the bulk, and at the endocardium, as opposed to the epicardium. As AF progresses, the asymmetry between epicardium and endocardium weakens and reentrant circuits move deeper into the bulk of the atrial wall. Hence, the average distance of reentrant circuits from the endocardial surface increases as AF becomes more persistent. As reentrant circuits move deeper into the atrial wall, they become harder to ablate.

3. Ablation in the model

We can simulate ablation in the model by permanently disabling a set of nodes corresponding to the desired ablation lesion (see Appendix C). In clinical practice, ablation is carried out by burning lesions from the endocardial surface.

By ablating active reentrant circuits in the model, AF can be terminated and the model will return to sinus rhythm. An example of this process is shown in Fig. 18. By ablating part of the reentrant circuit, the back-propagating excitation is blocked from reentering the excitable tissue and the fibrillatory activity dissipates.

In clinical practice, ablation lesions can typically only penetrate up to 2 mm into the atrial wall from the endocardial surface. The atria is 1–7 mm thick [14], averaging approximately 2.5 mm [55]. As a result, reentrant circuits which are far from the endocardium may be difficult to destroy using ablation. This is shown in Fig. 6 of the main paper.

4. Robustness of model results

The results presented in the main paper are for a tissue with specific thickness and a single choice of fiber orientations. Figure 3(b) is for the model with a thickness of 25 nodes and for fiber orientation parameters of $\Delta\theta_{\text{endo}} = 24^\circ$ at the endocardium ($z = 0$) and $\Delta\theta_{\text{epi}} = 42^\circ$ at the epicardium ($z = 24$). These values have not been chosen arbitrarily. The tissue thickness corresponds to the average atrial thickness noted in Ref. [55], and the values for the variation in fiber orientation are taken directly from the experimental studies on transmural reentrant circuits [14,16].

The core results presented in the main paper are robust against changes in the thickness of the tissue and against different choices of fiber orientation. To assess the robustness, we have tested the model for different tissue thicknesses and different values of the fiber orientation. Figure 19 shows the risk curves and associated driver depth distributions for the model for thinner (ten nodes, 1.0 mm) and thicker tissue (50 nodes, 5.0 mm), and for different choices of fiber orientation parameters on the endo- and epicardium.

The fiber orientation parameters tested cover a range of possible cases that might be relevant to the atria. Figures 19(a) and 19(b) correspond to strong longitudinal coupling on the endocardium, with uniform coupling on the epicardium. Figures 19(c) and 19(d) correspond to the moderate longitudinal coupling on the endocardium and almost uniform coupling on the epicardium (these parameters match those used in the main paper). Figures 19(e) and 19(f) correspond to very mild longitudinal coupling on the endocardium and very mild transverse coupling on the epicardium. Finally, Figs. 19(g) and 19(h) correspond to uniform coupling on the endocardium and strong longitudinal coupling on the

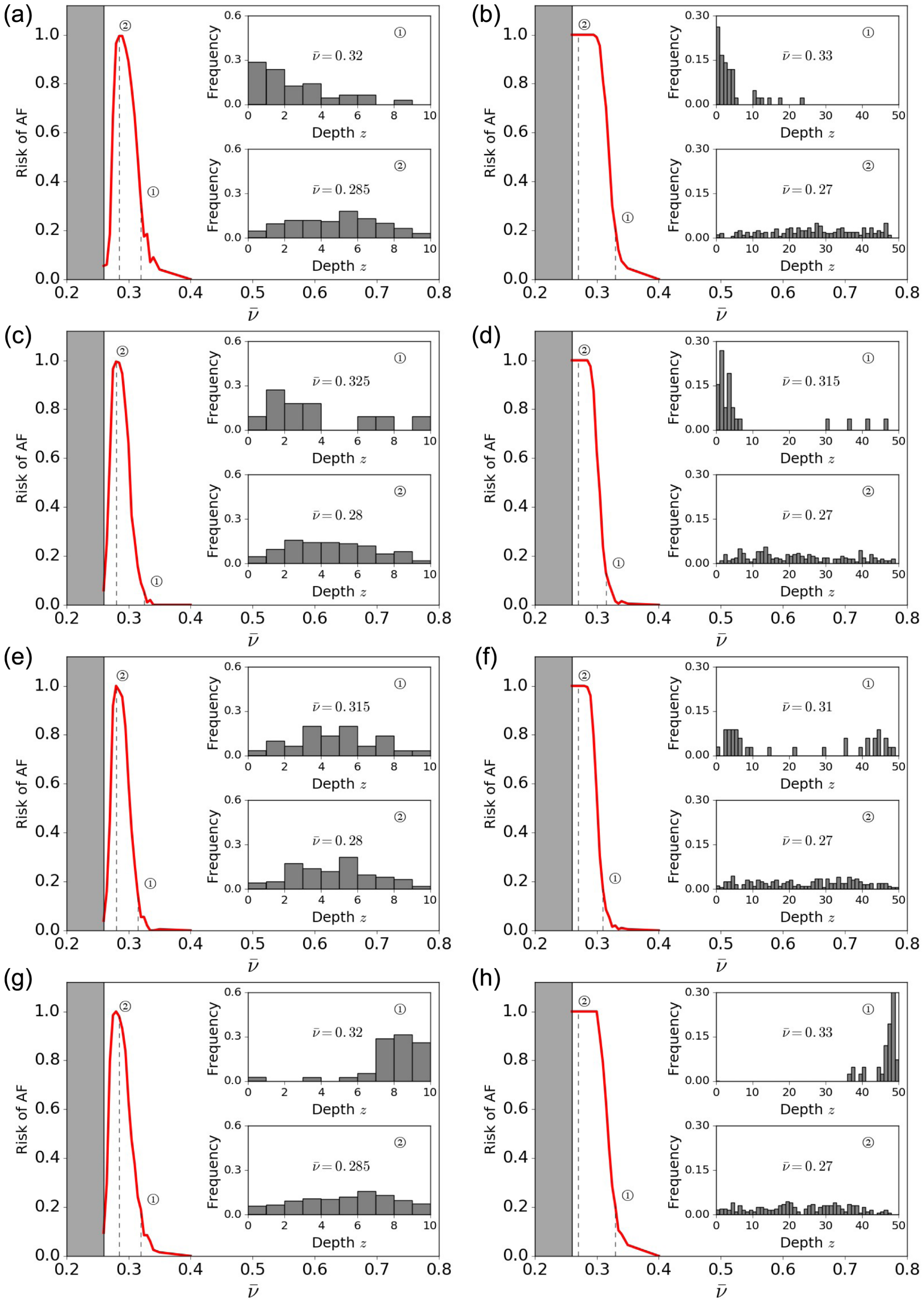

FIG. 19. Inhomogeneous coupling parameter risk curves (red graph) for various choices of fiber orientation and tissue thickness. Inset: Histograms showing the distribution of reentrant circuits driving AF for paroxysmal AF (top) and persistent AF (bottom) as a function of depth z from the endocardium. Left column: Tissue thickness of ten nodes (1.0 mm). Right column: Tissue thickness of 50 nodes (5.0 mm). (a, b) Strong longitudinal coupling on endocardium, $\Delta\theta_{\text{endo}} = 10^{\circ}$, and uniform coupling on the epicardium, $\Delta\theta_{\text{epi}} = 45^{\circ}$. (c, d) Moderate longitudinal coupling on endocardium, $\Delta\theta_{\text{endo}} = 24^{\circ}$, and nearly uniform coupling on epicardium, $\Delta\theta_{\text{epi}} = 42^{\circ}$. Parameters correspond to those used in Fig. 3(b) of the main paper. (e, f) Very mild longitudinal coupling on endocardium, $\Delta\theta_{\text{endo}} = 40^{\circ}$, and very mild transverse coupling on epicardium, $\Delta\theta_{\text{epi}} = 60^{\circ}$. (g, h) Uniform coupling on endocardium, $\Delta\theta_{\text{endo}} = 45^{\circ}$, and strong longitudinal coupling on epicardium, $\Delta\theta_{\text{endo}} = 10^{\circ}$. (g) and (h) are identical to (a) and (b) with the endocardium and epicardium swapped.

epicardium—the reverse case of Figs. 19(a) and 19(b). For models with no change in fiber orientation from endocardium to epicardium, see Fig. 16.

The results consistently show that reentrant circuits are predominantly found near the atrial wall with the strongest longitudinal coupling in the paroxysmal AF case ($\bar{\nu}$ is large). However, at lower absolute coupling ($\bar{\nu}$ is small), corresponding to the persistent AF case, circuits are distributed evenly between the endo- and epicardium. This result is more pronounced for thicker tissue than thinner tissue, since the spatial extent of circuits allows a circuit to anchor to both the endo- and epicardium if the tissue is too thin. These results indicate that the regions expected to be most at risk in clinical AF are those with strong longitudinal muscle fibers such as the pectinate muscles identified in Ref. [14]. A detailed discussion of the robustness of the 2D model can be found in Ref. [58].

APPENDIX E: VIDEO

Video of the activation patterns observed in the model can be found in the Supplemental Material [37]. Captions for the video are listed below. All video are for the inhomogeneous model where the average node-to-node coupling is fixed, $\bar{\nu} =$ constant, and variation in fiber direction varies linearly from $\Delta\theta_{\text{endo}} = 24^\circ$ at the endocardium ($z = 0$) to $\Delta\theta_{\text{epi}} = 42^\circ$ at the epicardium ($z = 24$). The model has dimensions $L_x = L_y = 200$ and $L_z = 25$ as outlined in the model definition in Appendix A. The remaining model parameters are $\delta = 0.05$, $\epsilon = 0.05$, $\tau = 50$, and $T = 220$.

1. Videos A

Video A features a close-up video of the endocardium and the epicardium showing the spontaneous emergence of AF in the inhomogeneous model, $\bar{\nu} = 0.31$. A reentrant circuit forms near the endocardial surface. This video corresponds to Figs. 1 and 4(b) in the main paper. The epicardial breakthrough is shown in Fig. 11(a).

2. Videos B

Video B features a close-up video of the endocardium and the epicardium showing the spontaneous emergence of AF in the inhomogeneous model, $\bar{\nu} = 0.33$. A reentrant circuit forms near the endocardial surface. This video corresponds to Fig. 14 in Appendix D. The epicardial breakthrough is shown in Fig. 11(b).

3. Videos C

Video C features a close-up video of the endocardium and the epicardium showing the spontaneous emergence of AF in the inhomogeneous model, $\bar{\nu} = 0.32$. A reentrant circuit forms near the endocardial surface. The epicardial breakthrough is shown in Fig. 11(c).

4. Videos D

Video D features a close-up video of the endocardium and the epicardium showing the spontaneous emergence of AF in the inhomogeneous model, $\bar{\nu} = 0.31$, and a cross section through the atrial wall showing the formation of a reentrant circuit near the endocardial surface and the propagation of fibrillatory waves through the atrial wall to the epicardium. The endocardial view in this video corresponds to Fig. 12(b). The cross section corresponds to Fig. 2 in the main paper.

5. Videos E

Video E features a close-up video of the endocardium and the epicardium showing the spontaneous emergence of AF in the inhomogeneous model, $\bar{\nu} = 0.32$. A reentrant circuit forms near the endocardial surface. The epicardial breakthrough is shown in Fig. 11(e).

6. Video F

Video F features a full scale video showing the simultaneous activation patterns on the endocardium and epicardium, and cross sections through the atrial wall, $\bar{\nu} = 0.32$. The video shows many of the key features of emergent AF in the model. The model starts in sinus rhythm before a single reentrant circuit forms. This circuit forms temporarily and dies out after one cycle. This temporary circuit forms three times before a new sustained reentrant circuit emerges near the endocardium. The new sustained circuit drives fibrillatory activity across the tissue blocking new wavefronts from entering the tissue at the pacemaker, $x = 0$. Shortly after time 2000, the reentrant circuit is ablated and fibrillatory waves dissipate. However, the interaction between the dissipating fibrillatory waves and a new pacemaker wavefront results in a new short lived circuit forming at time 2500. The new fibrillatory waves dissipate before the tissue returns to sinus rhythm. Note that the ablation lesion disrupts all future sinus rhythm wavefronts, although the effect is small.

7. Video G

Video G features a full scale video showing the emergence of AF before being terminated by ablation, $\bar{\nu} = 0.33$. The reentrant circuit forms near the endocardium before being destroyed by ablation. The fibrillatory waves dissipate before the tissue returns to sinus rhythm (see Fig. 18).
